# LGN Directs Interphase Endothelial Cell Behavior via the Microtubule Network

**DOI:** 10.1371/journal.pone.0138763

**Published:** 2015-09-23

**Authors:** Catherine E. Wright, Erich J. Kushner, Quansheng Du, Victoria L. Bautch

**Affiliations:** 1 Curriculum in Genetics and Molecular Biology, University of North Carolina, Chapel Hill, North Carolina, United States of America; 2 Department of Biology, University of North Carolina, Chapel Hill, North Carolina, United States of America; 3 McAllister Heart Institute, University of North Carolina, Chapel Hill, North Carolina, United States of America; 4 Lineberger Comprehensive Cancer Center, University of North Carolina, Chapel Hill, North Carolina, United States of America; 5 Department of Neurology, Institute of Molecular Medicine and Genetics, Georgia Regents University, Augusta, Georgia, United States of America; University of Illinois at Chicago, UNITED STATES

## Abstract

Angiogenic sprouts require coordination of endothelial cell (EC) behaviors as they extend and branch. Microtubules influence behaviors such as cell migration and cell-cell interactions via regulated growth and shrinkage. Here we investigated the role of the mitotic polarity protein LGN in EC behaviors and sprouting angiogenesis. Surprisingly, reduced levels of LGN did not affect oriented division of EC within a sprout, but knockdown perturbed overall sprouting. At the cell level, LGN knockdown compromised cell-cell adhesion and migration. EC with reduced LGN levels also showed enhanced growth and stabilization of microtubules that correlated with perturbed migration. These results fit a model whereby LGN influences interphase microtubule dynamics in endothelial cells to regulate migration, cell adhesion, and sprout extension, and reveal a novel non-mitotic role for LGN in sprouting angiogenesis.

## Introduction

Endothelial cells (EC) cooperate to form and maintain blood vessels, processes that are crucial developmentally and co-opted in numerous diseases [1, 2]. Formation of vessel networks requires intricate coordination of EC migration, adhesion, and polarization as EC undergo sprouting angiogenesis to form new conduits and expand vessel networks [3–5]. New sprouts form by re-orienting initiating cells, called “tip cells”, in the proximal-distal axis and by activation of pro-migratory pathways [6]. Migrating tip cells do not divide often, but stalk cells behind the tip cell in the sprout divide to maintain linkages as the sprout extends. EC divide with the division plane perpendicular to the proximal-distal axis of the sprout, which contributes to its lengthening [7]. Tip and stalk cells switch places periodically, indicating that dynamic cell rearrangements accompany blood vessel expansion [8].

Microtubules (MTs) are α/β tubulin polymers that form a network regulating cell morphogenesis, migration, and polarity [9, 10]. MT polymerization (growth) is countered by pauses, by activity of MT-severing factors, and by “catastrophe” that leads to rapid depolymerization (shrinkage), processes that collectively are termed dynamic instability. During interphase MTs originate from a centrosome-nucleated MT organizing center (MTOC) and from the Golgi, and they serve as tracks for motor-based transport of cargos to different cellular locations. MTs also interact with and regulate cell-cell adhesion junctions, and with focal adhesions that link cells to the surrounding matrix, in ways that are incompletely understood [11, 12]. MTs affect EC coalescence into tubes, branching morphogenesis, and mediate interphase effects of excess centrosomes on EC sprouting and migration [13–15].

LGN (Pins (Partner of Inscuteable), GPSM-2, (G protein signaling modulator 2)) is an adaptor protein that participates in MT-orienting complexes [**16**, 17]. LGN has 3 characterized domains: a GoLoco domain that interacts with the Gαi subunit of G-proteins in the GDP state, a linker domain, and a TPR domain that interacts with other proteins. The role of LGN in mitosis is well-studied, where it forms a complex with membrane-localized Gαi via its GoLoco domain and with NuMA, a protein that also interacts with the MT motor protein dynein/dynactin, via its TPR domain [18]. LGN is thought to be in a closed conformation during interphase via TPR-GoLoco interactions that are disrupted by NuMA after nuclear breakdown at the onset of mitosis. The Gαi-LGN-NuMA complex anchors astral microtubules and orients spindles. During mitosis, LGN also participates in a complex that involves the Par3-Par6-aPKC polarity proteins and Inscuteable (mInsc), a protein that interacts with MTs, perhaps via a kinesin [19–22]. How these complexes operate relative to each other and other polarity cues during mitosis is not well-understood. However, LGN is required for oriented divisions during *Drosophila* and mouse development and in polarized 3D models, and many LGN-regulated divisions are asymmetric and regulate stem cell production vs. differentiation *in vivo* [**16**, 17, 23–26]. Recent studies suggest that LGN also regulates aspects of cell polarity during interphase [27, 28], but how LGN affects interphase cells and whether this translates into effects on morphogenesis is unclear.

Here we investigated the role of LGN in angiogenic sprouting and EC behavior. We found that sprouting endothelial cells do not require LGN for spindle orientation, but LGN is required for proper EC sprouting and branching. EC behaviors important in sprout formation—migration, cell-cell adhesion, and cell-matrix adhesion—are affected in EC with reduced levels of LGN. We also observed effects of LGN manipulation on interphase MT dynamics. Since MT dynamics are upstream of cell adhesion and migration, we propose that LGN controls EC behaviors through the MT network during interphase, and that this novel non-mitotic function is important for proper angiogenic sprouting.

## Methods

### Cell Culture

Human Umbilical Vein Endothelial Cells (HUVEC) (Lonza) were cultured in EBM2 (Lonza) supplemented with EGM2 bullet kit (Lonza) and 1X Anti-Anti (Gibco), and used at passage 2–6. For starvation conditions, OptiMEM (Gibco) was supplemented with 0.5% fetal bovine serum (FBS, Gibco) and 1X Antibiotic-Antimycotic (Gibco, #15240). HEK293T (Clontech) and Normal Human Lung Fibroblasts (NHLF, Lonza) were cultured in DMEM with 10% FBS and 1% Anti-Anti and used at passage 4–12.

### RNA Knockdown

For lentivirus constructs, a tdTomato reporter was introduced into LGN KD (5’-GGTCTAAGCTACAGCACAAAT-3’) and EV constructs [18] at the GFP reporter site. Lentivirus was produced by the UNC Lentiviral Core. Additional LGN targeting constructs (TRCN0000011025 (5’-GCATGATTATGCCAAAGCATT-3’), TRCN0000006469 (5’-GCAGATACTATTGGAGATGAA-3’)) were obtained from Thermo Scientific. Targeting constructs were co-transfected with viral packaging plasmids pRSV Rev, pMDL RRE, and pVSV-g (Addgene) into HEK293T cells, and viral supernatants were collected 48 hr post transfection. Low passage (P2-3) HUVEC were infected overnight in supplemented EBM2 media with 10ug/mL polybrene. Infected HUVEC were assayed a minimum of 72 hr post-infection and used until P6.

For siRNA experiments, SilencerSelect Custom siRNA (Invitrogen, 5'-GGAAGUUAAGUGAAUCAUATT-3') or non-targeting (NT) controls were used. siRNA (10 nmoles) was transfected into cells using RNAiMax transfection reagent (Invitrogen) in antibiotic-free medium overnight. All assays were performed 48 hr after initial transfection.

### *In Vitro* Angiogenesis Assay

The sprouting angiogenesis assay was performed as described [29]. HUVEC were infected with virus 72 hr prior to the start of the assay. 10^6^ HUVEC were coated onto Cytodex microcarrier beads and allowed to settle overnight, then suspended in 2mg/mL fibrinogen (Sigma, Fisher) plus 0.15 units/mL aprotinin (Sigma) in PBS. Upon addition of 0.625 U/mL thrombin (Sigma), the fibrinogen clotted to form a fibrin matrix. NHLF were plated on top of the fibrin, and media (EBM2 supplemented with EGM2 bullet kit) was added and changed every second day.

### Random Migration Assay

HUVEC were sparsely plated on coverslips treated with 1ug/mL fibronectin (Sigma) 4 hr prior to imaging. Single cells expressing the virus-encoded reporter were selected, and images were acquired at 10 min intervals over 12 hr. Cells that migrated out of frame or divided were excluded. The center of the nucleus was followed, and migration coordinates were obtained using the Manual Tracking plug-in in FIJI and quantified in Excel.

### Immunofluorescence

Cultured HUVEC were fixed in 4% PFA for 10 min followed by 10 min permeabilization in PBS/0.5% Triton X-100. Sprouting HUVEC were fixed in 2% PFA for 20 min, followed by 2 hr incubation in PBS/0.5% Triton X-100. Samples were blocked in staining solution (PBS/0.5% Triton X-100/1% BSA/1% goat serum/0.2% sodium azide) for 2 hr at RT or overnight at 4°C. Primary antibodies (1:100) in stain solution were incubated at 4°C overnight. Samples were washed 3X 10 min in stain solution and incubated in Alexa fluor secondary antibodies (1:250, 1 hr at 37° for cultured HUVEC; 1:50 overnight at 4° for sprouting HUVEC). Phalloidin (1:50 in stain solution) was incubated overnight, and DAPI and DRAQ5 (1:5000, Invitrogen) were incubated 1 hr at RT. Conjugated phosphohistone H3 488/555 (rabbit polyclonal, Cell Signaling, 1:100) was incubated overnight at 4°C. VE-Cadherin (Enzo, rabbit anti-human) was incubated overnight at 4°C at 1:200.

### MT Nucleation Assay

HUVEC expressing control or LGN KD vectors were incubated in OptiMEM plus nocodazole (5ug/ml in DMSO; Sigma) for 3 hr at 37°C. Cells were rinsed 2X in cold OptiMEM, then incubated in EBM2 at 37°C and fixed in 100% cold MeOH for 2 min post-washout. Cells were stained with α-tubulin conjugated to Alexa Fluor 555 (rabbit monoclonal, 1:200, Millipore) as described.

For MT stability measurements, MT were depolymerized by placing HUVEC on ice for 20 min, then growth media at 37°C was added for 1 hr. For experiments requiring PHEM (60 mM PIPES, 25 mM HEPES, 10 mM EGTA, 2 mM MgSO4)/Triton-X100 treatment, the PHEM/Triton X-100 was at 37°C and added after growth media washout, immediately prior to fixation in cold MeOH. Slides were washed in PBS/0.01% Tween20 (PBST) 3X and blocked in 10% new born calf serum diluted in PBST (blocking buffer) for 2 hr. After blocking, primary antibodies: α-tubulin-555-conjugate 1:250 (Millipore), detyrosinated (deTyr) tubulin 1:500 (Sigma), or acetylated (Ac) tubulin 1:500 (Abcam), were added in blocking buffer and incubated overnight at 4°C. Slides were washed 3X in blocking buffer at RT, then incubated with secondary antibody in blocking buffer for 2 hr. Secondary antibodies were Alexa fluor conjugates (1:250, Invitrogen). Slides were washed in PBST at RT and mounted in Vectashield mounting media (Vector Labs) prior to imaging.

### EDTA Washout Assay

HUVEC were grown until completely confluent, then treated with EDTA (3mM final concentration) for 1 hr. Thereafter, cells were washed 4X with PBS and incubated in HUVEC media for 1 hr, then fixed and stained as indicated. VE-cadherin area was measured by imaging HUVEC monolayers. Images were converted to 8-bit depth and then thresholded (same thresholding criteria for all groups). Under high magnification a rectangular box was tiled to generate multiple independent measurements across a single confocal image. The percent area that was fluorescent generally encompassed 2–5 cells. The bounding box area was standardized between groups and measurements were expressed as average % VE-cadherin area. All analyses were performed on at least 5 random areas.

### Focal Adhesion Analysis

HUVEC were treated with nocodazole, incubated in EMB2 at 37°C for 20 min, then fixed in 2% PFA and stained with vinculin (mouse monoclonal, 1:100, Abcam). Fifteen images per condition were acquired. Static properties of focal adhesions were analyzed using FAAS (http://faas.bme.unc.edu/) with the following parameters: detection threshold 2, minimum adhesion size 2 pixels, and minimum FAAI ratio 3. Output was processed in Excel and Prism.

### PlusTip Tracking and Analysis

Cultured HUVEC were co-infected with control or LGN KD-tdTomato and EB1-GFP virus and imaged as described [15], using a PerkinElmer UltraView spinning disk confocal microscope with ORCA-ER camera, Nikon 60× Plan Apo NA 1.4 objective, and MetaMorph software. Briefly, images were captured at 2 sec intervals and the first 30 sec were analyzed. Analysis was done using plusTipTracker in MatLab [30].

### Imaging and Quantification

Cultured HUVEC were imaged on a Leica DMI 6000B or Olympus LSM5 confocal microscope. Sprouting HUVEC were imaged on an Olympus LSM5 confocal microscope. Live imaging of HUVEC was performed on an Olympus FV10 or Olympus VivaView microscope system. Images were processed in LSM Image Browser and FIJI with Manual Tracking, Metamorph, and Chemotaxis plug-ins. Quantification of cell detachment, sprout length, branchpoint frequency, and line scans were done in FIJI. Graphing and statistical analyses were done in Excel and Prism. All statistical comparisons were done using unpaired student’s t-test, two tailed, or one-way ANOVA with Tukey’s test, as indicated in the Figure Legends.

## Results

### Reduced LGN Perturbs Angiogenic Sprouting

**Fig 1:**
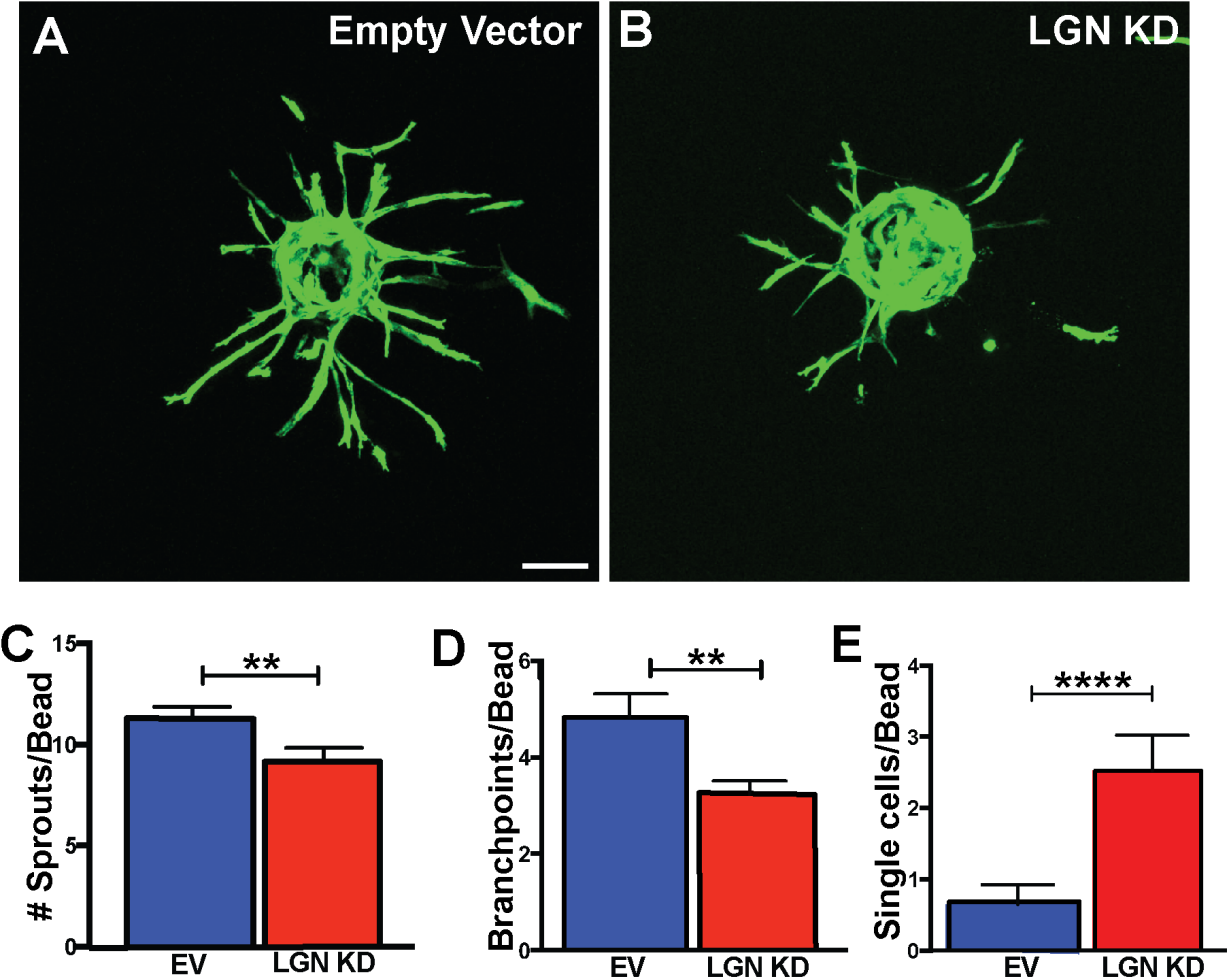
Loss of LGN perturbs sprouting angiogenesis. **(A, B)** Confocal images (compressed z-stacks) of representative beads with sprouting HUVEC and indicated viral infection. Green, GFP-labeled infected HUVEC. **(C-E)** Quantification of indicated parameters. Statistics, unpaired student’s t-test, two-tailed. Error bars, SEM; n = 5 experiments; **, p<0.01. ****; p<0.0001.

To explore the role of LGN during sprouting angiogenesis, we used a previously characterized LGN shRNA-expressing virus [18] to reduce LGN expression in EC. Infected HUVEC showed decreased LGN RNA levels by qRT-PCR analysis and protein by Western blot **(S1A and S1C Fig)**. We employed a sprouting angiogenesis assay [29] to determine effects of LGN knockdown (KD) in selected beads with at least 50% infected cells, as determined by GFP reporter expression. LGN KD HUVEC had significantly fewer sprouts compared to controls, and these sprouts displayed reduced branching **(Fig 1A–1D)**. We obtained two additional shRNAs against LGN that reduced LGN expression in HUVEC and also decreased angiogenic sprouting **(S1B, S1D and S1E Fig).** Taken together, these data suggests that LGN promotes sprout formation during angiogenesis.

Surprisingly, we observed that LGN KD EC were more likely to be present as single cells dissociated from a sprout **(Fig 1E and S1F Fig)**. We initially hypothesized that the dissociated EC resulted from failed branching and disconnection from the parent vessel. However, we found no correlation between the frequency of dissociated EC and branching frequency within the same bead **(S1G Fig)**, suggesting that the dissociated EC are not directly downstream of branching defects.

LGN contributes to proper spindle orientation during mitosis in epithelial tissues, so we asked whether the sprouting defects we observed resulted from disrupted mitotic orientation. However, in contrast to a requirement for LGN in oriented divisions in other epithelia [18], HUVEC spouts showed no significant changes in division orientation with LGN KD **(S1H and S1I Fig)** suggesting that LGN is dispensable for spindle orientation in endothelial sprouts.

### LGN Affects Endothelial Cell-Cell Adhesion

**Fig 2:**
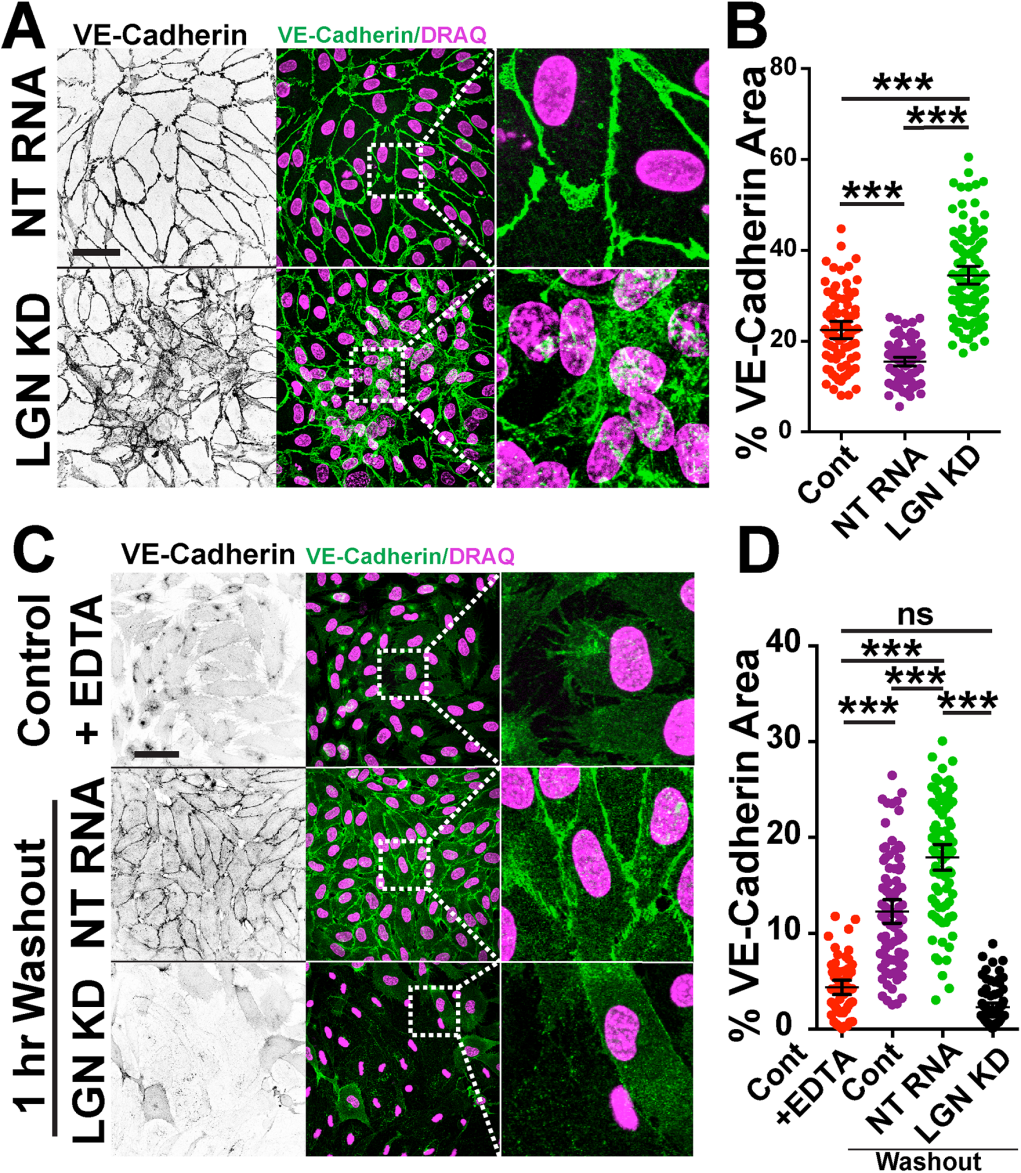
Cell-cell adhesions are destabilized in LGN KD HUVEC. **(A)** Representative images of HUVEC monolayers stained for VE-cadherin (green) and nucleus (DRAQ, pink) between indicated groups. **(B)** Scatter plot of % VE-cadherin area in HUVEC monolayers between indicated groups. Statistics, one-way ANOVA with Tukey’s test; n = 2 experiments; ***, p<0.001.**(C)** Representative images of HUVEC monolayers stained for VE-cadherin (green) and nucleus (DRAQ, pink) pre- and post- EDTA washout. After washout, junctions were allowed to reform for 1 hr. **(D)** Scatter plot of % VE-cadherin area in EC monolayers between indicated groups. White boxes, areas of higher magnification. Control, HUVEC; NT RNA, HUVEC +non-targeting RNA; LGN KD, HUVEC + LGN siRNA. Statistics, one-way ANOVA with Tukey’s test. Error bars, mean and 95% confidence intervals (CI); n = 2 experiments; ***, p≤ 0.001; ns, not significant. Scale bars, 10μm.

The finding that reduced LGN levels were accompanied by dissociated EC in vascular sprouts led us to investigate the effects of LGN on cell-cell junctions. VE-cadherin is a central component of EC adherens junctions, and junction stability often correlates with VE-cadherin localization, with stable junctions showing tight, thin staining at EC borders while destabilized junctions are associated with less junctional VE-cadherin and a more diffuse signal [3, 31]. VE-cadherin staining of HUVEC monolayers revealed that LGN KD cells had elevated area covered by the VE-cadherin signal compared to controls **(Fig 2A and 2B**), suggesting that reduced LGN leads to less stable, disorganized junctions with concomitant increase in non-junctional VE-cadherin. We next examined the ability of EC to reform cell-cell junctions after EDTA-mediated dissociation and washout. One hour after washout, junctions of LGN KD cells had not reformed to the levels of controls **(Fig 2C and 2D)**, supporting that junctions are not able to reform efficiently with reduced LGN. These data indicate that reduced LGN levels affect junction formation and stability.

### LGN Regulates Endothelial Cell Migration

**Fig 3:**
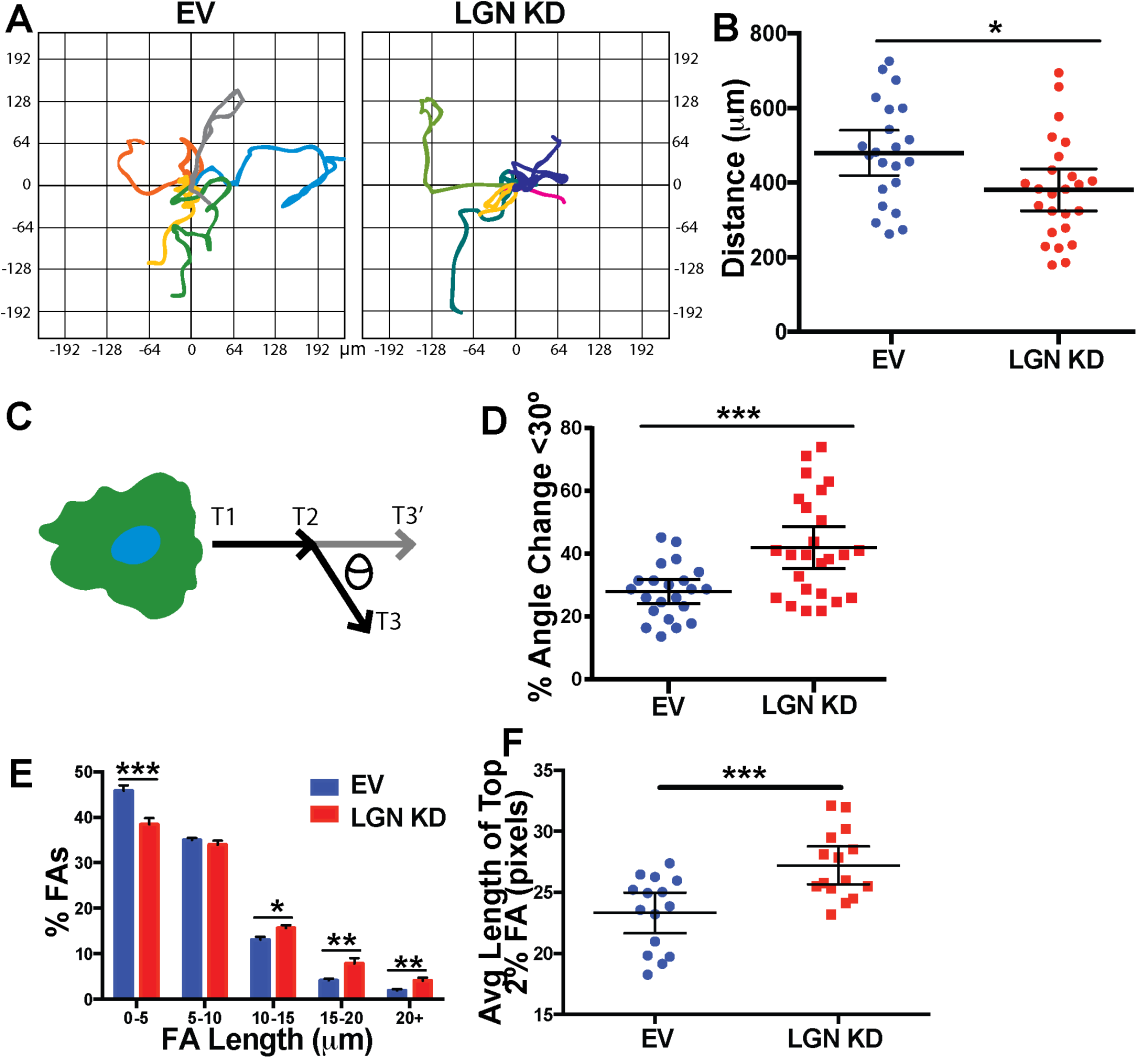
LGN KD HUVEC have reduced migration and perturbed focal adhesion turnover. **(A)** Plots of individual cell migration tracks, axes in μm. **(B)** Quantification of total distance traveled over 12 hr. Statistics, unpaired student’s t-test, two-tailed. Error bars, average and 95% CI; n = 3 experiments; *, p<0.05. **(C)** Schematic showing directional change measurement. **(D)** Percentage of EC angle changes that averaged less than 30° over 12 hr for a given cell. Statistics, unpaired student’s t-test, two-tailed. Error bars, average and 95% CI; n = 3 experiments; ***, p<0.001. **(E)** Distribution of focal adhesion (FA) length in control and LGN KD HUVEC 20 min after nocodazole washout. Statistics, unpaired student’s t-test, two-tailed. Error bars, SEM; n = 3 experiments; *, p<0.05; **, p<0.01; ***, p<0.001. **(F)** Scatter plot of longest FAs in control and LGN KD HUVEC 20 min after nocozadole washout. FAs were visualized by vinculin staining and analyzed using FAAS (http://faas.bme.unc.edu/) and parameters defined in Methods. Statistics, unpaired student’s t-test, two-tailed. Error bars, mean and 95% CI; n = 3 experiments; ***, p<0.001.

Angiogenesis requires effective migration to generate new sprouts, so we hypothesized that reduced LGN levels perturbed EC migration. We used live-imaging to assess migration in a random walk assay, and found that LGN KD HUVEC traveled significantly shorter distances compared to controls **(Fig 3A and 3B and S2A and S2B Fig),** suggesting that LGN influences cell motility. A component of cell migration is directional motion, so we asked whether LGN influenced the ability of EC to change direction. We calculated angle differences between directional vectors for individual movements between time-points [32], and found that reduced LGN levels significantly impaired the ability of HUVEC to make large (>30°) directional changes **(Fig 3C and 3D and S2C Fig)**. These data support a role for LGN in regulating the distance and direction of migration, and suggest that migration effects contribute to the perturbed angiogenic sprouting seen with reduced LGN.

Focal adhesions (FAs) provide anchors to the matrix, and FA turnover is necessary for cell migration. FA turnover rates can be determined by measuring FA regrowth after pharmacological dissociation, and longer FAs often correlate with reduced turnover. To determine whether reduced LGN levels affected FA dynamics, HUVEC were exposed to nocodazole to depolymerize MTs and halt FA turnover [33]. Assessment of FA length upon washout showed that LGN KD HUVEC had a higher frequency of long focal adhesions compared to controls (**Fig 3E and 3F)**. These results suggest that FA turnover is reduced in HUVEC with loss of LGN, and are consistent with the abnormal migration parameters.

### Loss of LGN Perturbs Microtubule Dynamics

**Fig 4:**
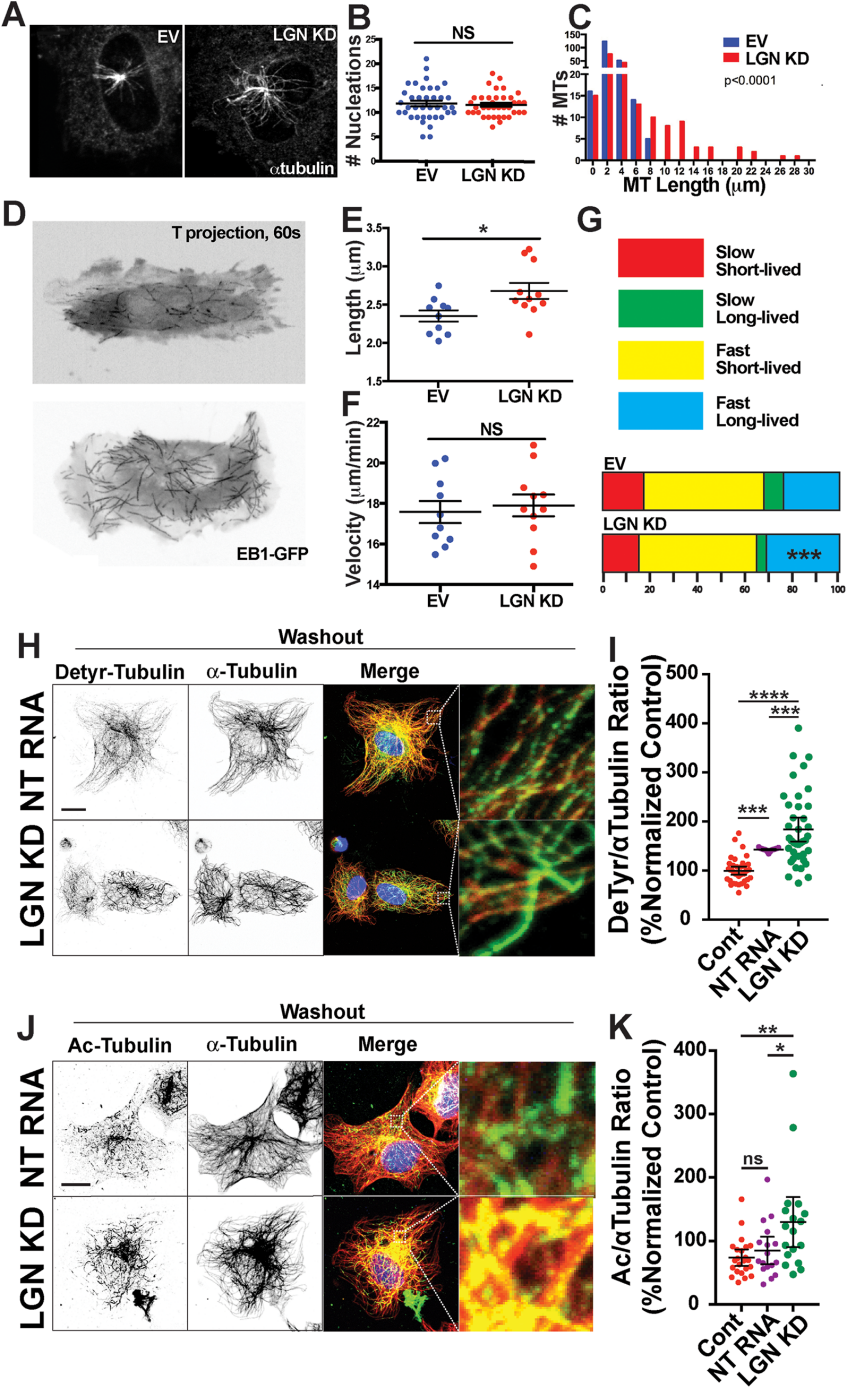
Abnormal MT dynamics and stability in HUVEC with reduced LGN. **(A)** Representative images of EV and LGN KD HUVEC stained with α-tubulin 1 min post-nocodazole washout. **(B)** Scatter plot of MT nucleations post-nocodazole washout. Statistics, unpaired student’s t-test, two-tailed. Error bars, mean and 95% CI; n = 3 experiments; NS, not significant. **(C)** Distributions of MT length 1 min post-nocodazole washout; Statistics, unpaired student’s t-test, two-tailed; n = 3 experiments; EV, n = 210; LGN KD, n = 188; p<0.0001. **(D)** Time projections of 60 sec movies of EB1-GFP labeled MT plus-ends in EV and LGN KD HUVEC. **(E-F)** Quantification of indicated parameters. EV, n = 10 cells; LGN KD, n = 11 cells. Statistics, unpaired student’s t-test, two-tailed. Error bars, mean and 95% CI; n = 2 experiments; *, p = 0.01; NS, not significant. **(G)** Distribution of MT plus ends based on lifetime and growth speed. Statistics, unpaired student’s t-test, two-tailed; n = 2 experiments; ***, p<0.001. **(H)** Representative images of HUVEC 1 hr post cold washout stained for MTs (α-tubulin, red), detyrosinated MTs (green), and nuclei (blue) between control (Cont); non targeting siRNA (NT RNA) and LGN KD siRNA treated cells. **(I)** Scatter plot comparing detyrosinated tubulin levels as a ratio of detyrosinated tubulin/α-tubulin between groups. Statistics, one-way ANOVA with Tukey’s test; n = 2 experiments; ***, p<0.001; ****, p<0.0001. **(J)** Representative images of HUVEC 1 hr post cold washout stained for MTs (α-tubulin, red), acetylated MTs (green), and nuclei (blue) between indicated groups. **(K)** Scatter plot comparing acetylated tubulin levels as a ratio of acetylated tubulin/α-tubulin between groups. White boxes, areas of higher magnification. Statistics, one-way ANOVA with Tukey’s test. Error bars, mean and 95% CI; n = 2 experiments; ns, not significant; *, p≤0.05; **, p≤0.01. Scale bars, 10μm.

Cell-cell adhesions, focal adhesions and migration, all processes perturbed in EC with reduced LGN levels, are regulated by interactions with MTs, and LGN participates in MT-mediated orientation during mitosis in other epithelia. Thus we hypothesized that LGN influences MT dynamics in interphase EC. Excess centrosomes can alter MT dynamics and EC migration [14]; however, centrosome numbers in interphase HUVEC with reduced LGN were not elevated **(S3A Fig)**. We next analyzed MT dynamics in a re-nucleation assay, and found that LGN KD HUVEC had similar levels of MT nucleation but significantly longer MTs compared to control HUVEC **(Fig 4A–4C)**. We hypothesized that the increased length of MTs upon re-nucleation in LGN KD HUVEC resulted from more rapid growth of MTs. To test this idea, we visualized MT dynamics via live-imaging of HUVEC infected with an EB1-GFP-expressing virus to label the plus-end of growing MTs [30]. Consistent with results of the re-nucleation assay, we observed significantly longer comets in LGN KD HUVEC compared to controls **(Fig 4D and 4E)**. Combined, these data indicate that LGN contributes to MT dynamics by regulating MT lengthening.

Microtubules in LGN KD EC are longer, which could either be due to an increased rate of MT polymerization or result from increased MT stabilization [9, 15]. Although we observed no global difference in comet velocity **(Fig 4F)**, binning of MTs by growth rate and lifetime revealed a significantly larger population of fast longer-lived MTs in LGN KD HUVEC **(Fig 4G)**, suggesting that LGN regulates MT stability and growth rate. MTs are often directionally polarized, with the new MT growth concentrated parallel to the migration vector. However, MT comet polarization was not significantly compromised in LGN KD HUVEC, suggesting that LGN does not influence MT polarity **(S3B Fig)**. To further examine MT stability, we examined expression of two modifications associated with stabilized MTs, detyrosination (deTyr) and acetylation (Ac). The ratios of tubulin with both modifications were significantly elevated in LGN KD EC compared to controls **(Fig 4H–4K)**, strongly suggesting that MTs are longer in part because they are more stabilized with reduced LGN levels. Taken together, these data indicate that LGN influences the dynamic instability necessary for MT growth and turnover, and that reduced levels of LGN compromise MT dynamics and lead to over-stabilization with consequent reduced FA turnover and impaired EC migration.

## Discussion

Here we show that LGN functions during angiogenesis to regulate blood vessel sprouting and branching. Manipulating LGN levels perturbs endothelial cell-cell and cell-matrix adhesion, likely downstream of changes in MT dynamics. These cellular effects are accompanied by abnormal EC migration, which is predicted to affect 3D sprouting. These findings identify a novel non-mitotic role for LGN in the regulation of EC behaviors and their organization into sprouts.

LGN functions in a complex with Gαi and NuMA during mitosis to capture astral microtubules and orient the spindle, leading to oriented cell divisions that are important for asymmetric cell differentiation and tissue morphogenesis [16–18, 34]. Although blood vessel endothelium is structurally organized as an epithelium, and other epithelia require LGN for oriented division, LGN is not required for oriented cell division in sprouting HUVEC. However, oriented endothelial cell division is important for proper vessel morphogenesis [7], suggesting that other mechanisms are dominant over Gαi-LGN-NuMA mediated positioning of astral microtubules in sprouting EC. One plausible idea is that the elongated cell shape of EC in 3D sprouts constrains placement of the spindle poles such that the spindle pole axis is parallel to the sprout axis. EC in sprouts are about 10 times longer in the proximal-distal axis (parallel to the sprout axis) relative to the perpendicular axis, and they are also very flat, with the apical (luminal) surface near the basal (ablumenal) surface. In contrast, epithelia that require LGN for spindle positioning have more equivalent lengths in the different axes, with substantial depth to the lateral edge. As described by Hertwig [35, 36] and shown in 2D [37], cell shape can dictate the division plane, and our data suggests that cell shape obviates a requirement for LGN acting in a mitotic polarity complex during angiogenic sprouting.

Although LGN does not affect mitosis, it is required for proper vascular sprouting and branching, and for normal EC migration. These findings reveal a novel non-mitotic role for LGN in angiogenic sprouting, and the migration defect is likely mediated by changes in MT dynamics. MTs are longer and more stable with reduced LGN levels, and these changes are predicted to affect the ability of a cell to regulate adhesions and change direction. EC with reduced LGN levels do not turn as strongly as controls in random walk assays, and FAs are longer, suggesting that they are more stable. These findings are consistent with reports that MT targeting of FAs promotes disassembly, while persistent MTs promote FA growth [38–40]. They are also consistent with a recent report that MT stabilization via taxol reduced cell migration parameters [41].

Vascular sprouting requires migration of EC that remain connected to neighbors via cell-cell junctions. Reduced LGN led to elevated numbers of EC that were not attached to sprouts, suggesting a defect in cell-cell junctions that was supported by analysis of VE-cadherin staining in 2D. These findings are also consistent with MT effects on the stability of adherens junctions. Induction of MT growth in confluent monolayers via EB3 perturbation leads to disassembly of junctions [42], and collective migration *in vivo* requires co-ordination of cell-cell junctions that are dependent on dynamic MTs [43]. Thus, our work suggests that MT growth dynamics depend on proper LGN levels, and reduced LGN promotes increased MT growth that destabilizes adherens junctions in 2D and leads to detachment in 3D.

It is not known how LGN acts to regulate EC adhesion and migration, although our data is consistent with an interphase role for LGN in MT dynamics. During mitosis, LGN acts as a linker by associating with NuMA that binds to the TPR domain of LGN, and with Gαi at the cortex that binds the GoLoCo domain of LGN [18]. This complex interacts with MTs via the minus-end motor complex dynein/dynactin. LGN also associates with an adapter, mInsc (Inscuteable), via the TPR domains, and mInsc in turn recruits the aPKC-Par3-Par6 polarity complex that is required for polarized migration [44]. This complex is thought to interact with MTs via discs large and the plus-end motor Khc73 [20, 45]. aPKC can also prevent LGN co-localization via phosphorylation of the linker region of LGN, leading to binding of 14-3-3 that inhibits LGN-Gαi interactions [46]. Although interphase LGN interactions are less understood, a recent report finds that LGN and a family member, AGS3, are required for directional migration of neutrophils in a gradient, via anterior anchoring with Gαi and recruitment of mInsc that in turn recruits the aPKC polarity complex [28]. Since neutrophil migration is MT-dependent [47, 48], these interactions are predicted to affect MTs indirectly. A more direct interaction between interphase LGN and MTs was dissected in cochlear hair cells, where Gαi3-mediated localization of mPins (LGN) is mutually exclusive with aPKC complex localization, and both complexes are thought to orient the primary cilium [27]. In this cell type, Gαi/mPins co-localized with the MT plus end protein EB-1 and was hypothesized to directly affect MT dynamics, perhaps by recruiting a MT depolymerase. Although exactly how LGN affects angiogenic sprouting remains to be elucidated, our finding that LGN functions during interphase in EC migration and sprouting angiogenesis suggests that novel pathways contribute to blood vessel formation, and reveal a novel non-mitotic function for LGN in EC.

## Supporting Information

S1 Fig shRNA/siRNA validation and HUVEC sprouting phenotypes. A-B) qRT-PCR, relative expression of LGN in HUVEC infected as indicated. Samples were normalized to TBP1. Statistics, one-way ANOVA with Tukey’s test relative to control or EV. Error bars, SEM; n = 3 experiments; *, p<0.05; ***, p<0.001.C) Western blot showing reduced LGN with siRNA KD. D-F) Quantification of indicated parameters per bead in control and shRNA virus-infected HUVEC. Statistics, one-way ANOVA with Tukey’s test. Error bars, SEM; n = 3 experiments; *, p<0.05; **, p<0.01; ns, not significant. G) Branch points per bead versus single cells per bead. Slopes were not statistically different (p = 0.70); n = 5 experiments. H) Angiogenic sprouts with LGN KD HUVEC; green, GFP reporter in virus; red, phosphohistone H3 staining (mitotic cells); white, phalloidin (actin cytoskeleton). White arrow, LGN KD cell in anaphase within a sprout; arrowhead, LGN KD cell in mitosis on the bead. I) Quantification of division angles relative to the long axis of the cell. Statistics, unpaired student’s t-test, two-tailed; n = 8 experiments; ns, not significant. EV, empty vector; LGN KD, LGN knockdown; NT RNA, non-targeting RNA.
(PDF)

S2 Fig LGN KD perturbs HUVEC migration. A) Plots showing individual cell movements over 12 hr. Axes in μm. B-C) Quantification of indicated parameters in control (EV, empty vector) and shRNA virus-infected (LGN KD2, LGN KD3) HUVEC. Statistics, one-way ANOVA with Tukey’s test. Error bars, mean and 95% CI; n = 3 experiments; *, p<0,05; **, p<0.01; ***, p<0.001.
(PDF)

S3 Fig LGN KD does not affect EC centrosome number or MT polarity. A) Quantification of excess centrosomes in HUVEC with indicated manipulations. Statistics, one-way ANOVA with Tukey’s test; n = 3 experiments; ns, not significant. Error bars, SEM. B) Rose plot of MT plus end growth angle distribution in HUVEC with indicated virus infection. Statistics, unpaired student’s t-test, two-tailed; n = 2 experiments; ns, not significant. EV, empty vector; LGN KD, LGN knockdown.
(PDF)
