# Supplementary material for: LGN Directs Interphase Endothelial Cell Behavior via the Microtubule Network
Source: PLoS One. 2015 Sep 23;10(9):e0138763. doi: 10.1371/journal.pone.0138763 (PMC4580422; doi:10.1371/journal.pone.0138763)
Supplement: S1 Fig — A-B) qRT-PCR, relative expression of LGN in HUVEC infected as indicated. Samples were normalized to TBP1. Statistics, one-way ANOVA with Tukey’s test relative to control or EV. Error bars, SEM; n = 3 experiments; *, p<0.05; ***, p<0.001.C) Western blot showing reduced LGN with siRNA KD. D-F) Quantification of indicated parameters per bead in control and shRNA virus-infected HUVEC. Statistics, one-way ANOVA with Tukey’s test. Error bars, SEM; n = 3 experiments; *, p<0.05; **, p<0.01; ns, not significant. G) Branch points per bead versus single cells per bead. Slopes were not statistically different (p = 0.70); n = 5 experiments. H) Angiogenic sprouts with LGN KD HUVEC; green, GFP reporter in virus; red, phosphohistone H3 staining (mitotic cells); white, phalloidin (actin cytoskeleton). White arrow, LGN KD cell in anaphase within a sprout; arrowhead, LGN KD cell in mitosis on the bead. I) Quantification of division angles relative to the long axis of the cell. Statistics, unpaired student’s t-test, two-tailed; n = 8 experiments; ns, not significant. EV, empty vector; LGN KD, LGN knockdown; NT RNA, non-targeting RNA. (PDF) [file pone.0138763.s001.pdf]

# Supporting Information

## SUPPLEMENTAL FIGURE 1

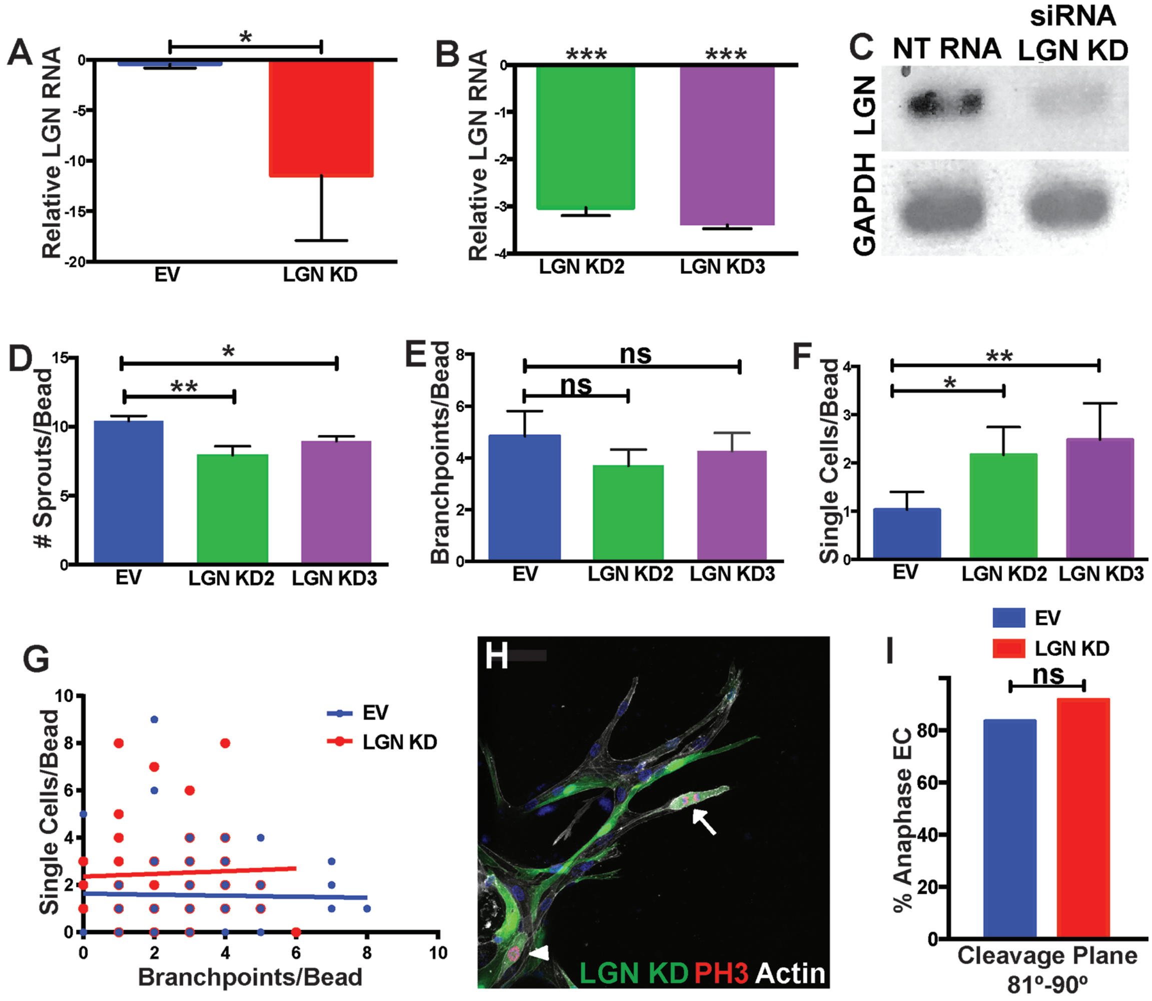


**Figure S1. shRNA/siRNA validation and HUVEC sprouting phenotypes.** A-B) qRT-PCR, relative expression of LGN in HUVEC infected as indicated. Samples were normalized to TBP1. Statistics, one-way ANOVA with Tukey's test relative to control or EV. Error bars, SEM; n=3 experiments; *, $p<0.05$; ***, $p<0.001$.C) Western blot showing reduced LGN with siRNA KD. D-F) Quantification of indicated parameters per bead in control and shRNA virus-infected HUVEC. Statistics, one-way ANOVA with Tukey's test. Error bars, SEM; n=3 experiments; *, $p<0.05$; **, $p<0.01$; ns, not significant. G) Branch points per bead versus single cells per bead. Slopes were not statistically different ($p=0.70$); n=5 experiments. H) Angiogenic sprouts with LGN KD HUVEC; green, GFP reporter in virus; red, $\alpha$-phosphohistone H3 staining (mitotic cells); white, phalloidin (actin cytoskeleton). White arrow, LGN KD cell in anaphase within a sprout; arrowhead, LGN KD cell in mitosis on the bead. I) Quantification of division angles relative to the long axis of the cell. Statistics, unpaired student's t-test, two-tailed; n=8 experiments; ns, not significant. EV, empty vector; LGN KD, LGN knockdown; NT RNA, non-targeting RNA.
