# Supplementary material for: LGN Directs Interphase Endothelial Cell Behavior via the Microtubule Network
Source: PLoS One. 2015 Sep 23;10(9):e0138763. doi: 10.1371/journal.pone.0138763 (PMC4580422; doi:10.1371/journal.pone.0138763)
Supplement: S2 Fig — A) Plots showing individual cell movements over 12 hr. Axes in μm. B-C) Quantification of indicated parameters in control (EV, empty vector) and shRNA virus-infected (LGN KD2, LGN KD3) HUVEC. Statistics, one-way ANOVA with Tukey’s test. Error bars, mean and 95% CI; n = 3 experiments; *, p<0,05; **, p<0.01; ***, p<0.001. (PDF) [file pone.0138763.s002.pdf]

SUPPLEMENTAL FIGURE 2

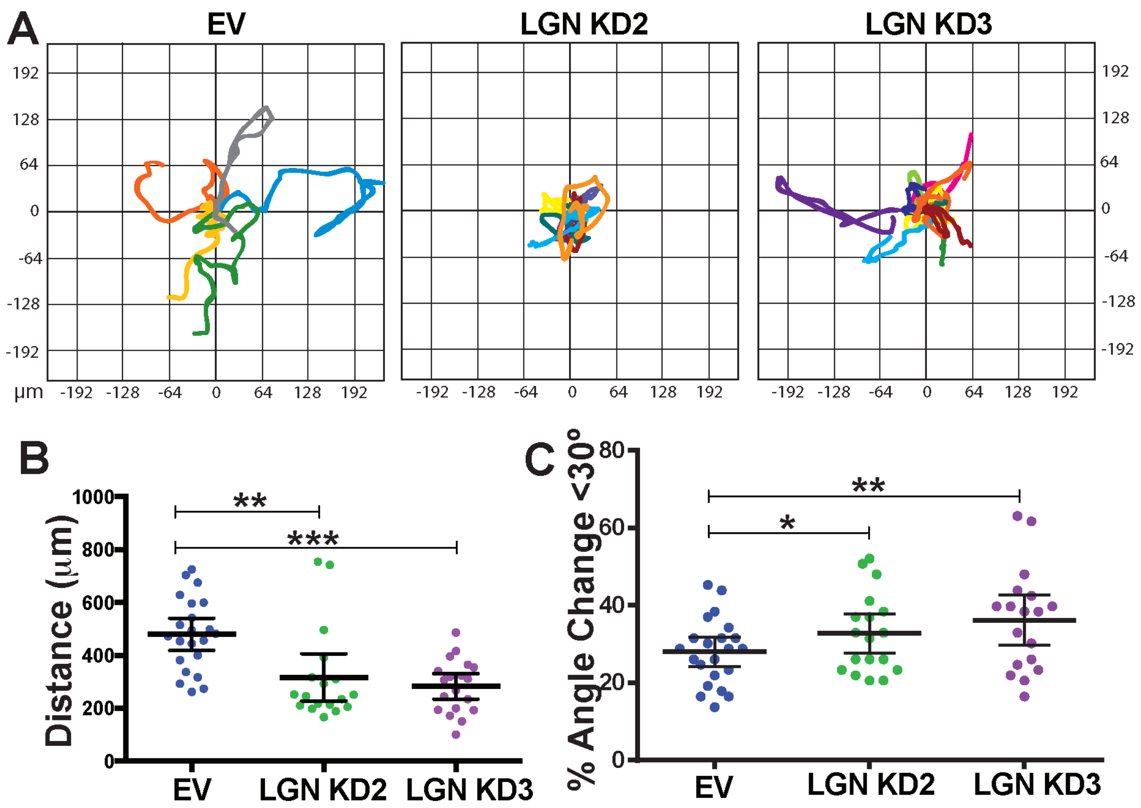

Figure S2. LGN KD perturbs HUVEC migration. A) Plots showing individual cell movements over 12 hr. Axes in µm. B-C) Quantification of indicated parameters in control (EV, empty vector) and shRNA virus-infected (LGN KD2, LGN KD3) HUVEC. Statistics, one-way ANOVA with Tukey's test. Error bars, mean and 95% CI; n=3 experiments; *, p<0,05; **, p<0.01; ***, p<0.001.
