# Supplementary material for: LGN Directs Interphase Endothelial Cell Behavior via the Microtubule Network
Source: PLoS One. 2015 Sep 23;10(9):e0138763. doi: 10.1371/journal.pone.0138763 (PMC4580422; doi:10.1371/journal.pone.0138763)
Supplement: S3 Fig — A) Quantification of excess centrosomes in HUVEC with indicated manipulations. Statistics, one-way ANOVA with Tukey’s test; n = 3 experiments; ns, not significant. Error bars, SEM. B) Rose plot of MT plus end growth angle distribution in HUVEC with indicated virus infection. Statistics, unpaired student’s t-test, two-tailed; n = 2 experiments; ns, not significant. EV, empty vector; LGN KD, LGN knockdown. (PDF) [file pone.0138763.s003.pdf]

SUPPLEMENTAL FIGURE 3

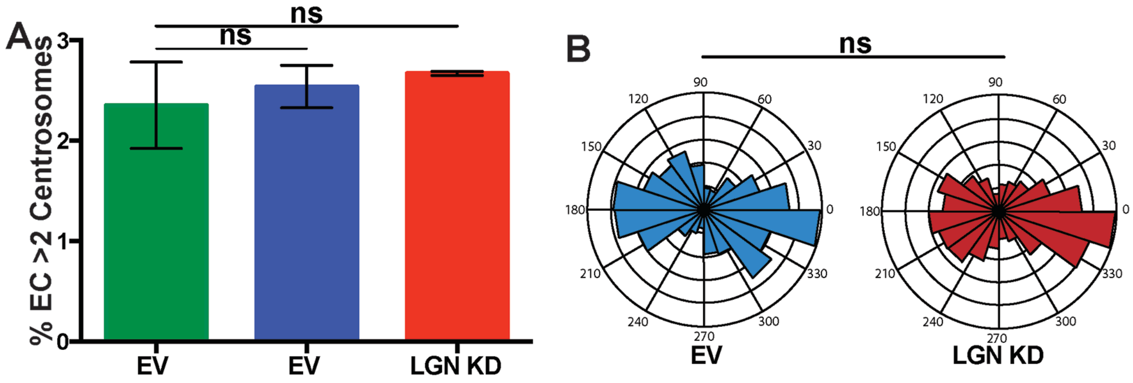

Figure S3. LGN KD does not affect EC centrosome number or MT polarity. A) Quantification of excess centrosomes in HUVEC with indicated manipulations. Statistics, one-way ANOVA with Tukey's test; n=3 experiments; ns, not significant. Error bars, SEM. B) Rose plot of MT plus end growth angle distribution in HUVEC with indicated virus infection. Statistics, unpaired student's t-test, two-tailed; n=2 experiments; ns, not significant. EV, empty vector; LGN KD, LGN knockdown.
